# Epidemiology of Child Maltreatment during the COVID-19 Pandemic in Saudi Arabia

**DOI:** 10.3390/children9030312

**Published:** 2022-02-24

**Authors:** Shuliweeh Alenezi, Mahdi A. Alnamnakani, Mohamad-Hani Temsah, Rozan Murshid, Fahad Alfahad, Haitham Alqurashi, Hana Alonazy, Mohamad Alothman, Majid Aleissa

**Affiliations:** 1 Department of Psychiatry, College of Medicine, King Saud University, Riyadh 11451, Saudi Arabia; fahadbalfahad@gmail.com; 2 Department of Psychiatry, King Saud University Medical City, King Saud University, Riyadh 12372, Saudi Arabia; 3 SABIC Psychological Health Research and Applications Chair (SPHRAC), Department of Psychiatry, College of Medicine, King Saud University, Riyadh 11451, Saudi Arabia; 4 Pediatric Department, College of Medicine, King Saud University, Riyadh 11451, Saudi Arabia; malnamnakani@ksu.edu.sa (M.A.A.); mtemsah@ksu.edu.sa (M.-H.T.); roza1066@gmail.com (R.M.); hana_k111@hotmail.com (H.A.); 5 Department of Psychiatry, Children’s Hospital, Ministry of Health, Taif 26514, Saudi Arabia; har59y@windowslive.com; 6 Pediatric Emergency Department, King Saud University Medical City, King Saud University, Riyadh 12372, Saudi Arabia; maalothman@ksu.edu.sa; 7 National Family Safety Program, King Abdulaziz Medical City, Ministry of National Guard Health Affairs, Riyadh 11426, Saudi Arabia; maleissa@yahoo.com; 8 Pediatric Department, College of Medicine, King Saud Bin Abdulaziz University for Health Sciences, Riyadh 11426, Saudi Arabia

**Keywords:** emotional maltreatment, physical maltreatment, neglect, sexual abuse, COVID-19, Saudi Arabia

## Abstract

Child maltreatment, especially during health crises, is a major public health issue transcending cultural, social, and racial contexts. We assessed the sociodemographic and related risk factors associated with the types and rates of child maltreatment. We also assessed the economic, social, and environmental characteristics of child maltreatment victims and their perpetrators, as they were reported to the Saudi National Family Safety Program (NFSP), with consideration of the COVID-19 pandemic’s impact. A secondary data analysis of a retrospective review was conducted to compare types and rates before and during the COVID-19 outbreak, utilizing descriptive and multivariate analyses on anonymized data from the NFSP. According to a predetermined list of relevant risk factors for child maltreatment outlined by the NFSP, these anonymized data were obtained and analyzed with no exclusion criteria (*n* = 1304). The findings showed that a child’s age correlated significantly and positively with their odds of being physically maltreated; as a child’s age increased by one year, on average, their corresponding predicted odds of being physically maltreatment tended to rise by a factor equal to 7.6% (*p* < 0.001). Neglected children, compared to those who had not been previously neglected, were predicted to be almost twice (2.23 times more) as likely to be victims of physical maltreatment on average (*p* < 0.001). Children were notably more likely to experience sexual abuse during the COVID-19 pandemic than those exposed to abuse during the period before (1.69 times). The COVID-19 pandemic was associated with significantly lower odds of physical child maltreatment (47.7% less). This study found no statistically significant effect of the COVID-19 pandemic on children’s odds of being emotionally maltreated (*p* = 0.169). These findings support the existence of specific risk factors for child maltreatment for both child victims and perpetrators. They also attest to the significant differences between different types of maltreatment. A systematic, proactive system is needed to screen and document child maltreatment with a higher degree of integration with community reporting systems.

## 1. Introduction

Globally, child maltreatment is a major health concern with an uncertain prevalence. The recognition of the importance of identifying, describing, and documenting suspected and confirmed cases of child maltreatment has been increasing both nationally and internationally [1]. The United Nations Children’s Fund (UNICEF), the World Health Organization (WHO), and other multinational agencies have asked that child maltreatment be acknowledged as a global public health crisis and have suggested strategies to address it [2]. Likewise, Saudi Arabia has become more aware of child maltreatment, as the public has sought to better understand this phenomenon. Reports indicate that children are subjected to physical punishment with varying degrees of severity. These acts of maltreatment, possibly performed as acts of child discipline, are engendered by a society that values child obedience and the positive effects of discipline [3]. However, the successful implementation of an intervention system, which includes child protection centers at medical facilities and required reporting and data gathering measures, has contributed to an increasing awareness of child maltreatment and abuse in Saudi Arabia. Moreover, the shift in public perception toward a better understanding of child maltreatment has aided in the detection and reporting of neglect [4].

Many nations have report greater demand for domestic violence services and higher risks of maltreatment in children who are not in school, a trend that is comparable to what has occurred in the aftermath of prior bouts of social isolation caused by diseases and pandemics [5]. When children are isolated at home, they are more likely to be maltreated. In addition, exposing children to family violence, whether direct or indirect, is damaging and can result in traumatic stress disorders and other severe emotional and behavioral problems [6]. In addition, child maltreatment and domestic abuse frequently coexist. Domestic abuse is linked to several variables during pandemics, including financial stress, family-dynamic instability, higher exposure to damaging relationships, and fewer support alternatives, all of which are crucial to consider as the COVID-19 pandemic unfolds [7]. Following natural disasters, Campbell [8] discovered that resources were quickly depleted. Maltreatment risk factors have also been identified as controlling behaviors (often used to cope with trauma), unemployment, and restricted access to support networks, all of which are common following natural disasters. The American Academy of Pediatrics and many health bodies worldwide have released new recommendations for increased support for vulnerable children of maltreatment [9].

According to recent research, the COVID-19 pandemic amplified some risk factors for child maltreatment. The economic activity was constrained as lockdown measures were tightened, resulting in income loss and unemployment for many people. People’s mental health has deteriorated due to restrictions on mobility and economic downturn [10]. A study based on survey data from 600 parents in Hong Kong found that the present epidemic has resulted in parental income loss and job loss, considerably increasing the likelihood of physical maltreatment of their children [11]. Similarly, another looked at survey data from 283 persons in the United States and found that the COVID-19 pandemic enhanced parents’ feelings of social isolation and job loss, leading to child neglect and verbal maltreatment [12]. Despite the lack of data, media reports and studies from governments and organizations suggested that domestic abuse, a risk factor for child maltreatment, surged as the COVID-19 pandemic was unfolding. For example, Zhang found that during the COVID-19 pandemic, police reports on domestic abuse incidents, primarily directed at women, increased by double to quadruple in several rural areas of China compared to the same period in 2019 [13].

At the local level in Saudi Arabia, child maltreatment is still a problem, and its prevalence is a source of concern for both government and healthcare organizations [14]. Some reports have indicated that the number of reported maltreatment cases is substantially lower than the absolute number of occurrences, either to the victim’s innocence or the investigating authorities’ callousness and insensitivity [15]. In response, National Family Safety Program (NFSP) was launched in late 2005 to help prevent child maltreatment and provide public awareness and education [16]. In addition, the COVID-19 pandemic has added another layer of complexity to the landscape of child maltreatment reporting, with more incidents of emotional maltreatment or sexual abuse than physical maltreatment or neglect [17].

In this study, we were keen on assessing the sociodemographic and related predisposing factors of child maltreatment cases reported by the NFSP’s data, assuming a difference in certain predisposing sociodemographic and environmental factors for both victims and perpetrators during the COVID-19 pandemic.

## 2. Methodology

The research team acquired approval from King Saud University’s (KSU) Institutional Review Board (IRB) to complete a retrospective review 12 months review of the NFSP of anonymized data. The NFSP is a centralized electronic system established and maintained under the National Guard Health Affairs (NGHA). Our study team received formal authorization from the NGHA for accessing the anonymized registry based on having received IRB approval. However, the NFSP fulfills the mandate for health institutions to document substantiated or unsubstantiated cases of maltreatment. The Ministry of Human Resources and Social Affairs is responsible for documenting community-reported incidents; these data are not included in our analysis.

The current study’s main aim is to report the rates and types of child maltreatment and the economic, social, and environmental characteristics associated with child maltreatment victims and perpetrators. With no exclusion criteria, we secured data for the period between September 2019 and September 2020 (N = 1304). Data collected according to a predetermined list of relevant risk factors for child maltreatment, as designed by an NFSP data collection form. The impact of the COVID-19 pandemic was also used as a constant factor to assess its impact on sociodemographic factors and types of maltreatment, using 23 March 2020 as our benchmark date, when Saudi Arabia’s first countrywide lockdown was issued.

Measures:

1. Victim demographic data characteristics: The investigating agency registers the victim’s gender, age, and city, alongside the educational and employment condition of the parents, as well as whether or not they are living together. The health status of the parents, as well as the presence of disabilities, are also documented.
2. Specifications about the maltreatment and the perpetuator: This part narrates the victim’s health preceding the incident,, and whether the victim has a chronic illness or disabilities. It also contains detailed about who reported the incident and if there is prior maltreatment exposure.
3. Type of maltreatment: A list with four choices is used (physical, emotional, sexual, and neglect). This section requires the identification of the offender’s link to the victim and additional information such as age, gender, marital status, educational attainment, job, general health, evidence of chronic illness and/or disability, and addiction.
4. The victim and perpetrator’s financial and environmental characteristics: This part enables agencies to determine characteristics utilizing a three-parts checklist:
  - Victim’s family socioeconomic characteristics: This section includes poor parenting, wrong social parenting-specific beliefs, parental divorce, social isolation, living in a high-crime district, a lack of supportive resources, insufficient household income, and/or residing in a remote area.
  - Victim-related variables: Excessive crying, chronic illness, history of neglect, history of maltreatment, established handicap, behavioral difficulties, and/or unwanted children are all documented in this section.
  - Perpetuator-related factors: Chronic medical conditions, poverty, unemployment, psychological and/or mental disorders, substance abuse and/or addiction, ignorance, and/or incorrect attitudes about offense and punishments are all included in this section.
5. Disposition decision: The investigating agency documents the healthcare outcome and whether the victim must be admitted (to the pediatric ward or PICU) or just a hospital or emergency department visit. It also includes referral plans for either the child-protection committee or law involvement.

## 3. Analysis

Continuous variables were subjected to a descriptive analysis using the mean and standard deviation, while categorical variables were subjected to frequencies and percentages. To control for the possible exposure to more than one form of maltreatment, multiple response dichotomy analysis was employed. It also was used to describe the prevalence of different types of maltreatment and associated factors. Based on the victims’ parents’ educational and work status, a standardized socioeconomic educational (SES) index score was calculated using factor analysis. In neglect cases where both parents are considered offenders (N = 103), we opted not to perform subsample analysis to simplify the analysis model.

Explanatory multivariate logistic regression was applied to assess the statistical significance of various predictors for multiple types of maltreatment among children. The selection of predictor variables and risk factors was based on the clinical, psychological, time-related, and sociodemographic relevance for each predictor to the subtypes of maltreatment being analyzed. The association between predictors with children’s risk of having been exposed to various types of maltreatment is represented as an odds ratio (OR) with a 95% confidence interval. The SPSS IBM V21 statistical data analysis program was used, and a 0.050 alpha significance level was considered.

## 4. Results

### 4.1. Sample Characteristics

Table 1 displays the victims’ sociodemographic characteristics and percentages based on date of incident.

Table 2 displays the yielded statistical analysis findings for the victims’ assault types and their offense types and the perpetrator’s personal characteristics. A quarter of the sample (25%) were identified to have been previously exposed to some form of maltreatment. The overall mean number of types of maltreatment experienced by these children was equal to 1.21 types per child on average (SD = 0.52), suggesting that some of the children may have experienced one or more types of maltreatment combined in one incident.

Table 3 displays the victims’ and their perpetrators’ family- and environment-associated factors and the victims’ final medical disposition.

### 4.2. Physical Maltreatment

The yielded analysis model (Table 4) showed that the period during which the COVID-19 pandemic spread across the globe was associated with significantly lower odds of child physical maltreatment; maltreated children were, on average, significantly less likely (47.7% less) to be physically maltreated during pandemic time compared to the non-pandemic period. Children who were confirmed for maltreatment at the point of contact with medical professionals were, in fact, significantly more (2.90 times more) likely to be physically maltreated than others whose physical symptoms showed probable markers of maltreatment (*p* < 0.001). Additionally, brothers were found to be significantly more likely to physically maltreated children (4.71 times more) on average compared to other offending persons (*p* < 0.001).

**Figure 1:**
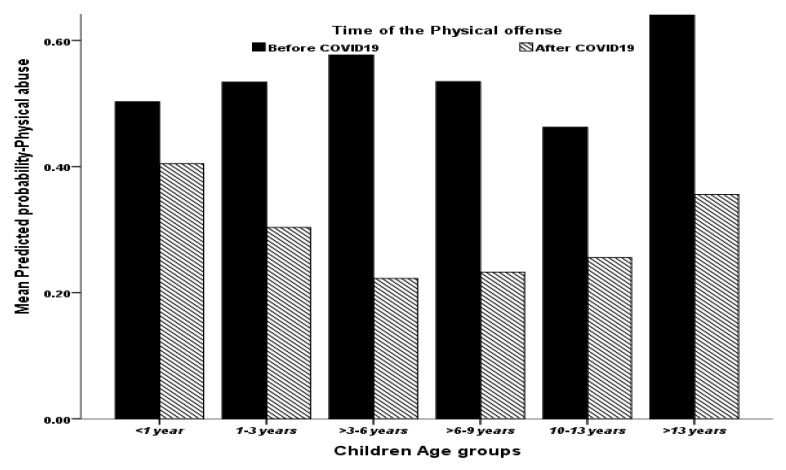
The association between children’s age group and their mead model-predicted probability of being physically maltreated.

The victim’s age correlated significantly and positively with their odds of being physically maltreated; as their age rose by one year on average, their corresponding predicted odds of being physically maltreated tended to rise by a factor of 7.6% (or 1.076 times more) on average as well (*p* < 0.001) (note Figure 1).

### 4.3. Sexual Abuse

Table 5 displays the results of the multivariate logistic binary regression analysis of reported sexual abused cases. The children were significantly more likely to experience sexual offense during the COVID-19 pandemic compared to those who were exposed to maltreatment in the period before. Furthermore, the odds of children being sexually abuse abused during the pandemic were 1.69 times higher on average. The children with known perpetrators were found to be significantly less (96.2% times less) likely to experience sexual abuse compared to those abused by an unknown offender (*p* < 0.001). In addition, children abused by males were found to be 5.80 times more likely to be sexually abused compared to children abused by females (*p* < 0.001). Additionally, the children abused by a single (unmarried) perpetrator were found to be significantly more likely (6.33 times more) to be sexually abused than other children abused by a married perpetrator or divorced perpetrators (*p* < 0.001). Moreover, children abused by perpetrators with psychological disorders were, on average, significantly more likely (3.16 times more) to experience sexual abuse compared to children who were abused by people who were mentally healthy (*p* < 0.001).

### 4.4. Emotional Maltreatment

The yielded analysis of the children’s odds of being emotionally maltreated (Table 6) showed that there was no statistically significant impact of the COVID-19 pandemic on the children’s odds of having been emotionally maltreated (*p* = 0.169). However, the pandemic appears to have been associated with slightly raised odds of emotional maltreatment in children, as well as in consideration of other independent predictor variables in the analysis model.

Children with behavioral problems were found to be significantly more likely (2.47 times more) to experience emotional maltreatment than those children without behavioral problems on average (*p* < 0.001). Moreover, the analysis model also showed that married and divorced offenders were significantly more associated with child emotional maltreatment (*p* < 0.001). Additionally, children living with both parents were found to be significantly less likely (77.2% less) to be emotionally maltreated compared to children living with a single parent or those living with other carers (*p* < 0.001).

### 4.5. Neglect

The multivariate analysis results (Table 7) showed that pandemic period was associated with significantly greater odds (45.7% higher) of neglect among children in the analyzed sample compared to the previous year (2019) on average (*p* = 0.014), accounting for other predictors in the analysis.

Children maltreated by married offenders were found to be significantly more likely (2.57 times more) to be neglected compared to children maltreated by single or separated offending persons on average (*p* < 0.001). However, single-parent children were found to be especially less likely (61.5% less) to be neglected compared to those residing with both parents and other careers (*p* < 0.001). Additionally, the analysis findings showed that divorced offenders were significantly more likely (5.41 times more) to neglect a child than other offending persons (*p* < 0.001). However, children living in socially isolated families were found to be significantly more (3.86 times more) likely to be neglected on average compared to children whose families were not socially isolated on average (*p* < 0.001).

## 5. Discussion

### 5.1. Physical Maltreatment Risk Factors

Our results are similar to those of other studies [18], which revealed an association between an increase in the incidence of physical maltreatment in children and an increase in their age. Our findings indicated that, as a child’s age rises by one year, the corresponding predicted odds of being physically maltreated increases by a factor of 7.6% on average. Moreover, evidence suggests that presence of chronic illnesses in children enhance the likelihood of physical abuse [19]. This suggests that certain characteristics unify this group of children, regardless of disability type or severity degree. However, a combination of age with specific socioeconomic characteristics is important. On the contrary, our results showed that children with chronic illness were significantly less likely to be physically maltreated compared to children who were not chronically ill. The study that identified the link between chronic illness in children and the increased risk of physical maltreatment involved a cross-sectional community survey, which is different from our hospital-based sample. However, we observed that children who have a brother experienced significantly higher levels of sibling violence [20].

### 5.2. Sexual Abuse Risk Factors

Our findings indicated that the victim’s sex was not a predictive factor of sexual abuse, which contradicts the findings of other studies that showed that female gender was a risk factor for sexual abuse and that female children were more prone to being sexually abused [21,22]. Moreover, children who live with a single parent are more prone to being victims of sexual abuse than children who live with two parents or other guardians. Therefore, protective measures must be implemented to target children who live in single-parent households (Carey et al., 2007). The literature shows that children with learning or physical disabilities are at a greater risk of sexual abuse [23]. We found a similar pattern between children with chronic medical conditions and/or disabilities and an increased risk of sexual abuse.

Murray, Nguyen, and Cohen [24] reported that children who were victims of sexual abuse were likely to live in homes in which the family was experiencing financial problems. On the contrary, children who were neglected probably lived in homes in which financial problems were present. Moreover, a review of multiple studies conducted in Africa by Meinck, Cluver, Boyes, and Mhlongo [25] uncovered the mother’s education being below a university education as a risk factor for the level of sexual abuse.

Data published by Meinck Cluver, and Boyes [26] showed a positive correlation between sexual abuse and household size; in their review, these authors found that families containing many people were associated with sexual abuse, whereas our results indicated that household size is not a significantly predictor of sexual abuse. Similar to our results, a paper published by McElroy and colleagues [27] showed high rates of neglect in sexually abused children. Additionally, they suggested that sexual abuse is accompanied by other forms of abuse, such as neglect, which matches the results of our analysis that indicated a child who was neglected before is more likely to be a victim of sexually abuse.

In terms of the offender’s characteristics, we found that being male is a predictable risk factor, which is similar to the findings of multiple studies [28]. In addition, our findings showed that most male perpetrators were strangers, compared to female offenders, who were mainly caretakers. In our study, the majority were male and strangers. Additionally, a study by Cullen, Hull Smith, Funk, and Haaf [29] comparing sexual abuse perpetrators and perpetrators of other crimes found that sexual abuse offenders were significantly more likely to be married than other perpetrators. Furthermore, a meta-analysis by Whitaker et al. (2008) was published comparing sex perpetrators against children and non-sex perpetrators that indicated that sex offenders have a greater history of mental illness compared to non-sex offenders [30].

### 5.3. Emotional Maltreatment Risk Factors

The age of the victim was found to be associated with emotional abuse, which implied that age may determine hesitancy or fear to seek help; older children may not receive help because they think it is too late, given their age [31]. Children who have been maltreated can become anxious, hostile, impulsive, and furious because they cannot cope with the stress. These reactions can lead to behavioral problems, such as aggressiveness, which can cause the parents to abuse the child even more. Thus, this correlates with our findings that behavioral problems are a risk factor of emotional maltreatment. Being married can also have an impact on emotional maltreatment, as women who were married against their will in an arranged marriage were more likely to maltreatment their children, and those who were maltreated as children may grow up to abuse their own children. Children who live with married parents are less likely to be emotionally maltreated, whereas those who live with a single parent, or one parent and a stepparent, are at a greater risk of being emotionally maltreated. Emotional neglect was more common among victims in cases in which the parents were the perpetrators, whereas physical and sexual abuse were more common when others were the perpetrators [32,33].

### 5.4. Neglect Risk Factors

Younger children were more prone to neglect in our study, which is similar to what was previously reported before the pandemic [34]. This is also similar to the findings of other studies that reported that infants and toddlers were more likely to suffer from neglect [35]. This is probably due to them being more dependent on their caregivers, which resulted in the infants and toddlers becoming more vulnerable to neglect during the crisis [36].

Our results also showed that children with chronic illnesses were significantly more likely to experience neglect. Berliana et al. (2019) reported that children with low- and high-severity disabilities were more likely to be neglected than non-disabled children, ranging from 3 to 11 times more, respectively. This situation may have been exaggerated during the pandemic, as the lockdown may have affected access to medical care for chronic illnesses. Therefore, care for very vulnerable, disabled children should be emphasized during any public health crisis.

Children living in socially isolated families or lacking social support services were found to be significantly more likely to be neglected. Plenty of evidence exists linking social isolation and limited social bonds with higher risks of child neglect [37]. This has even been demonstrated in several cultural contexts. On the contrary, large households with more than seven members were found to be significantly more likely to experience neglect in our results. Although the incidence of child neglect was highest for children who had a large family in several studies [38,39], others showed no correlation (Berliana et al., 2019).

### 5.5. Impact of COVID-19 on Different Types of Maltreatment

Since our data collection covered the period during the pandemic, it was intriguing to examine and report the effect of this international disaster on the different types of maltreatment. It has been reported that measures that were applied internationally to control the spread of the COVID-19 pandemic led to what was labeled a “secondary pandemic” among children, as evolving pictures of child neglect and abuse were anticipated during the crisis [40]. Addressing these changes requires more insight into each risk of child abuse, especially those present or amplified during the pandemic.

Our findings showed that tendencies toward increased vulnerabilities for physical maltreatment-related factors have existed during the COVID-19 pandemic. Many child welfare groups worldwide are reporting a significant decline in child abuse or neglect reports, which is consistent with our findings [8]. Children’s socio-ecological systems are disrupted in pandemic contexts, and as a result, the rate of child maltreatment is expected to rise [41]. Furthermore, during periods of parental burnout, an increase in children’s behavior problems may provoke harsh physical responses [42]. Children with special educational needs are also in danger; the disturbance of their daily routines may cause them to become angry and irritable [43]. To enable children in distress to call out for support, national helplines, guidance counselors, and other children-friendly reporting systems have been proposed globally. These resources must be tailored to the COVID-19 challenges [44].

However, sexual abuse seems to share more of a linear relationship with the COVID-19 pandemic. For example, the Uganda Child Helpline (UCHL) official reports about sexual abuse toward children reflected an increase in sexual abuse since the COVID-19 pandemic lockdown, representing the third most reported type of abuse, because the UCHL was the primary channel for reporting while schools and other facilities were closed. The majority of victims reported were females, and 17% of the perpetrators were family members [45].

In terms of other types of maltreatment, such as neglect and emotional maltreatment, the COVID-19 pandemic has not had a statistically significant effect on children’s odds of being emotionally maltreated (Campbell, 2020). However, the period during the pandemic appears to have been associated with slightly increased rates of emotional maltreatment in children, considering the other predictor independent variables in the analysis model. On the contrary, data reported during the pandemic were associated with significantly (45.7%) greater odds of neglect among children in the analyzed sample.

## 6. Conclusions

Child maltreatment continues to be a significant health issue in regular times. However, emphasis should be placed on its importance during national emergencies and disasters. Predictors for child maltreatment differ significantly based on the type of maltreatment. They seem to be influenced by cultural and reporting systems elements. Also, contexts linked with pandemics generate conditions in which children’s socio-ecological systems are severely impacted, increasing the likelihood of child maltreatment. However, our study found a significant decrease in child maltreatment reports during the COVID-19 pandemic, which was alarming. During the COVID-19 pandemic, there were also tendencies toward greater vulnerability to physical maltreatment-related variables. Sexual abuse, on the other hand, appears to have a more direct link to the COVID-19 pandemic. We noticed that the COVID-19 pandemic has had no statistically significant effect on children’s likelihood of being emotionally maltreated. On the opposite, data reported during the pandemic showed significantly greater odds of neglect among children.

## 7. Limitations

The NFSP data is limited to cases that have been presented to the healthcare system. Another community monitoring system is administered by the Ministry of Human Development and Social Affairs. As a result, our findings can be used to assess the influence of the COVID-19 health crisis on child maltreatment in Saudi Arabia for individuals who seek medical help.

Another limitation is the data’s cross-sectional nature, which only represents the first few months after the COVID-19 outbreak was declared and may not reflect the pandemic’s long-term influence on the Saudi population’s maltreatment picture. Furthermore, since this study used healthcare data solely, the impact of the lockdown on accessing healthcare could have an impact on the number of cases reported and hence the types of child maltreatment documented.

## 8. Recommendations

COVID-19 has altered the dynamics of child maltreatment in Saudi Arabia. To address these shifts, a better understanding of each risk of child maltreatment is required, especially those present or amplified during the pandemic. National helplines, counselors, and other child-friendly reporting mechanisms have also been recommended around the world to empower children at risk of maltreatment to seek help. These tools must be specifically adapted and tailored to the COVID-19 situational challenges and made accessible to Saudi Arabian children.

Another recommendation is to create a proactive system for screening for child maltreatment, particularly during times of crisis. Screening for child maltreatment requires a systematic, proactive tool with higher integration and optimization with community reporting mechanisms. The NFSP is now monitoring cases presented to healthcare facilities and other healthcare institutions, while the Ministry of Human Development and Social Affairs focuses on community monitoring and reporting. However, better collaboration between governmental agencies and more enhanced record integration is needed to create a comprehensive dynamic system.

## Figures and Tables

**Table 1:** Descriptive analysis of the sociodemographic characteristics of the victims of child maltreatment in Saudi Arabia.

	Frequency	Percentage
**Victim’s sex**		
Male	700	53.7
Female	604	46.3
**Victim’s age (Years), mean (SD)**		6.40 (4.88)
Age group		
<1 Year	98	7.5
1–3 Years	401	30.8
>3–6 Years	239	18.3
>6–9 Years	185	14.2
10–13 Years	250	19.2
>13 Years	131	10
**Residence**		
Eastern provinces	490	37.6
Western provinces	381	29.2
Northern provinces	51	3.9
Southern provinces	167	12.8
Central region	215	16.5
**Victim lives with**		
Both parents	821	63
Other caregivers	16	1.2
Other relatives	41	3.1
**Single parent**	426	32.7
**Father alive**		
No	18	1.4
Yes	1286	98.6
**Father has a chronic illness**		
No	1194	91.6
Yes	110	8.4
**Father has a disability**		
No	1291	99
Yes	13	1
**Father’s educational attainment**		
Unknown	38	2.9
Illiterate	49	3.8
Elementary school	118	9
Intermediate school	161	12.3
High school	690	52.9
University degree or higher	248	19
**Father’s employment**		
Unknown	40	3.1
Unemployed	100	7.7
Retired	111	8.5
Private sector	210	16.1
Governmental sector	756	58
Owns business	87	6.7
**Mother alive**		
No	15	1.2
Yes	1289	98.8
**Mother has a chronic illness**		
No	1230	94.3
Yes	74	5.7
**Mother has a disability**		
No	1295	99.3
Yes	9	0.7
**Mother’s educational attainment**		
Unknown	38	2.9
Illiterate	75	5.8
Elementary school	173	13.3
Intermediate school	190	14.6
High school	542	41.6
University degree or higher	286	21.9
**Mother’s employment status**		
Unknown	37	2.8
Unemployed	15	1.2
Housewife/retired = 1	1075	82.4
Private sector	51	3.9
Governmental sector	118	9
Owns business	8	0.6
**Date of accident**		
Before COVID-19	699	53.6
After COVID-19	605	46.4

**Table 2:** Descriptive analysis of the measured assault types and offending persons.

	Frequency	Percentage
**Reporting person**		
A stranger	11	0.8
A family member	608	46.6
The affected victim themselves	59	4.5
A healthcare worker	666	51.1
**Victim’s general health/condition before the incident**		
Healthy	1193	91.5
With a chronic illness/disability	111	8.5
**Possibility of maltreatment**		
Unsubstantiated case	404	31
Substantiated case	900	69
**Previous exposure to assault**		
No	978	75
Yes	326	25
**Type of previous assault**		
Neglect	176	54.3
Emotional maltreatment	59	18.2
Physical maltreatment	156	48.1
Sexual maltreatment	26	8
Other types	2	0.6
**Type of current assault**		
Sexual abuse	165	12.7
Emotional maltreatment	275	21.1
Physical maltreatment	541	41.5
Neglect	581	44.6
Other maltreatments	19	1.5
**Mean number of assault types/persons**		1.21 (0.52)
**Is the perpetrator known?**		
No	160	12.3
Yes	1144	87.7
**Offender**		
Unidentified person	160	12.3
Stepmother	16	1.2
Stepfather	12	0.9
Brother	40	3.1
Maternal uncle	20	1.5
Paternal uncle	16	1.2
Other relative	44	3.4
Stranger	80	6.1
Housemaid	10	0.8
Teacher	6	0.5
Mother	534	41
Father	470	36
**Offender’s sex**		
Female	504	38.7
Male	535	41
Both male and female	103	7.9
Unknown	162	12.4
**Offender’s marital status**		
Unknown	207	15.9
Single	119	9.1
Separated/divorced/widowed	266	20.4
Married	712	54.6
**Offender’s age group**		
≤18 Years	62	5.7
19–30 Years	379	34.9
31–40 Years	372	34.3
41–50 Years	204	18.8
51–60 Years	53	4.9
>60 Years	15	1.4
**Offender’s educational attainment**		
Illiterate	57	5.1
Primary school	150	13.5
Intermediate school	190	17.1
High school	524	47.1
University degree or higher	191	17.2
**Offender’s employment**		
**Both parents involved in offense**	103	7.9
Unemployed/student	134	10.3
Retired	51	3.9
Housewife	415	31.8
Private sector employee	103	7.9
Governmental employee	251	19.2
Owns business	33	2.5
Unknown	214	16.4
**Offender’s medical condition**		
Unknown	257	19.7
Healthy	948	72.7
With a chronic illness/disability	52	4
Known for addiction/substance use	47	3.6

**Table 3:** Descriptive analysis of the victims’ and perpetrators’ economic-, social-, and environment-associated factors.

	Frequency	Percentage
**Victim’s family socioeconomic factors**		
Large household size	122	9.5
Parental divorce	291	22.7
Other family-related problems	293	22.8
Social isolation	68	5.3
Poor parenting	745	58
**Victim’s environmental factors**		
Wrong social parenting-specific beliefs	796	61.6
living in a high-crime district	7	0.5
No supportive resources	35	2.7
insufficient income	135	10.4
Residing in a remote area	24	1.9
Unaware of the rules and laws	471	36.5
Other environment factors	306	23.7
**Victim-related characteristics, *n* = 1291**		
Child-related characteristics	392	30.4
Excessive crying	27	2.1
Child with chronic medical illness	87	6.7
history of neglect	543	42.1
Previously harmed	335	25.9
A disabled child	26	2
Child with behavioral problems	289	22.4
An unwanted child	26	2
A maltreated child	207	2
**Perpetuator-related factors/motives, *n* = 1296**		
Substance abuse/addiction disorders	60	4.6
Chronic medical illness	22	1.7
Other factors	350	27
unemployed	34	2.6
Has a psychological and/or psychiatric problems	123	9.5
Very young age	95	7.3
poverty	29	2.2
offense and punishment ignorance/wrong beliefs	771	59.5
**Hospital visit outcome**		
Hospital morgue	1	0.1
General hospital wards	367	28.1
Pediatric ICU	96	7.4
Emergency room	840	64.4
**Disposition outcome**		
Referral to child-protection team	905	69.7
Referred to police/law enforcement	323	24.9
Referred to other services	332	25.6
Required further medical assessment	12	0.9

**Table 4:** Multivariate logistic binary regression analysis of children’s odds of physical maltreatment (*n* = 1304).

	Multivariate Adjusted Odds Ratio (OR)	95% CI for OR		*p*-Value
		Lower	Upper	
Period of the incident = 2020 (during the COVID-19 pandemic)	0.523	0.390	0.702	<0.001
Victim’s sex = female	1.078	0.816	1.423	0.597
Victim’s age (years)	1.076	1.042	1.112	<0.001
Victim’s health condition = with chronic illness	0.321	0.185	0.556	<0.001
Victim’s household socioeconomic and educational index (factor score)	1.039	0.890	1.214	0.628
Previously neglected child	2.233	1.614	3.089	<0.001
Victim previously exposed to maltreatment	1.447	1.023	2.045	0.036
Large household size (large family size) = yes	0.555	0.330	0.933	0.026
Known offender to the victim/family	0.458	0.292	0.717	0.001
Male offender	1.513	1.075	2.130	0.018
Maltreatment possible = confirmed	2.857	2.027	4.028	<0.001
Victim with a behavioral problem	0.538	0.34	0.85	0.008
Parental divorce	1.722	1.196	2.478	0.003
Residing in remote place = yes	0.267	0.091	0.779	0.016
Offender’s ignorance/lack of awareness about punishment	1.514	1.106	2.074	0.010
Offender known to have a substance abuse issue	0.474	0.246	0.912	0.025
Excessive child crying	2.583	1.028	6.489	0.044
Disabled child	3.192	1.183	8.611	0.022
Offender is the brother	4.709	2.100	10.559	<0.001
Offender is a relative	0.227	0.077	0.672	0.007
Constant	0.185			<0.001

Dependent variable = physical maltreatment of the child (no/yes). The model’s overall statistical significance *χ*^2^(22) = 494.4, *p* < 0.001, AUC/ROC = 83.8%, and H–L goodness-of-fit *χ*^2^(8) = 18.3, *p* = 0.019.

**Table 5:** Multivariate logistic binary regression analysis of children’s odds of sexual abuse (*n* = 1304).

	Multivariate Adjusted Odds Ratio (OR)	95% CI for OR		*p*-Value
		Lower	Upper	
Period of the incident = 2020 (during the COVID-19 pandemic)	1.687	1.057	2.692	0.028
Victim’s sex = female	1.177	0.770	1.799	0.450
Victim’s age (years)	1.069	1.019	1.120	0.006
Living father = yes	0.379	0.093	1.553	0.178
Victim’s medical condition before the incident = with a disability/chronic disease	0.063	0.008	0.479	0.008
Victim’s household socioeconomic status and educational index (factor score)	1.383	1.075	1.779	0.012
Previously neglected child = yes	0.390	0.241	0.632	<0.001
Large household size (large family size) = yes	1.431	0.721	2.839	0.306
Family social isolation	1.204	0.487	2.976	0.688
Victim lives with a single parent (male/female)	1.840	1.151	2.941	0.011
Victim lives in eastern provinces = yes	0.160	0.079	0.326	<0.001
Known offender = yes	0.038	0.016	0.086	<0.001
Male offender	5.770	2.878	11.565	<0.001
Single (unmarried offender)	6.302	3.661	10.849	<0.001
Stranger offender	11.812	5.762	24.214	<0.001
A psychologically disordered offender	3.159	1.667	5.986	<0.001
Constant	0.562			0.459

Dependent variable = sexual abuse of the child (no/yes). The model’s overall statistical significance *χ*^2^(16) = 386.2, *p* < 0.001, AUC/ROC = 90%, and H–L goodness-of-fit *χ*^2^(8) = 2.30, *p* = 0.971.

**Table 6:** Multivariate logistic binary regression analysis of children’s odds of emotional maltreatment (*n* = 1304).

	Multivariate Adjusted Odds Ratio (OR)	95% CI for OR		*p*-Value
		Lower	Upper	
Period of the incident = 2020 (during the COVID-19 pandemic)	1.335	0.884	2.015	0.169
Victim’s sex = female	2.230	1.110	4.481	0.024
Victim’s age (years)	1.124	1.061	1.191	<0.001
Interaction (victim’s sex (female)	0.935	0.866	1.008	0.081
Victim’s household socioeconomic and educational index (factor score)	1.113	0.925	1.339	0.258
Maltreatment possibility = confirmed	0.419	0.275	0.637	<0.001
Victim is a child with known behavioral problems	2.465	1.581	3.842	<0.001
Parental divorce = +Ve	1.517	0.980	2.348	0.062
Offending person’s marital status = married	3.231	2.032	5.138	<0.001
Offending person’s marital status = divorced	2.901	1.733	4.858	<0.001
Father with a chronic illness	1.722	0.977	3.035	0.060
Mother with a chronic illness	2.352	1.229	4.502	0.010
Social isolation	2.338	1.191	4.587	0.014
History of poor parenting	1.426	0.962	2.112	0.077
History of previous neglect	2.334	1.430	3.807	0.001
Living with both parents	0.228	0.141	0.368	<0.001
Constant	0.026			<0.001

Dependent variable = emotional maltreatment of the child (no/yes). The model’s overall statistical significance *χ*^2^(18) = 471.23, *p* < 0.001, AUC/ROC = 88%, and H–L goodness-of-fit *χ*^2^(8) = 13.8, *p* = 0.088.

**Table 7:** Multivariate logistic binary regression analysis of children’s odds of neglect (*n* = 1304).

	Multivariate Adjusted Odds Ratio (OR)	95% CI for OR		*p*-Value
		Lower	Upper	
Period of the incident = 2020 (during COVID-19 pandemic)	1.457	1.079	1.970	0.014
Victim’s sex = female	1.048	0.795	1.381	0.741
Victim’s age (years)	0.910	0.881	0.940	<0.001
Victim’s household socioeconomic and educational index (factor score)	0.873	0.735	1.035	0.118
Victim’s health condition before assault = chronic illness	2.078	1.099	3.928	00.024
Mother with a chronic illness	0.398	0.193	0.821	0.013
Disabled mother	4.949	0.814	30.089	0.082
Married offender	2.566	1.754	3.756	<0.001
Living with a single parent	0.385	0.264	0.561	<0.001
Offender’s marital status = divorced	5.407	3.341	8.751	<0.001
Offending person is psychologically disordered	0.563	0.335	0.945	0.030
Family social isolation	3.857	1.913	7.778	00.000
Victim’s large household size (large family size) = yes	1.907	1.135	3.205	0.015
Offending person is very young	1.889	1.068	3.340	0.029
Victim is a child with a behavioral problem	0.396	0.253	0.618	<0.001
Child’s excess crying	0.148	0.054	0.411	<0.001
Child with a chronic disability	3.827	1.786	8.203	0.001
Environmentally high crime rate in the living area	6.762	1.011	45.226	0.049
Lack of social supportive services	4.302	1.702	10.877	0.002
Other environmental factors	3.259	2.340	4.538	<0.001
Constant	0.798			0.395

Dependent variable = neglect of the child (no/yes). The model’s overall statistical significance *χ*^2^(23) = 511.5, *p* < 0.001, AUC/ROC = 84%, and H–L goodness-of-fit *χ*^2^(8) = 43.15, *p* < 0.001.

## Data Availability

All of the data for this study will be made available upon reasonable request.
